# Supplementary material for: Age-related DNA methylation changes are sex-specific: a comprehensive assessment
Source: Aging (Albany NY). 2020 Dec 3;12(23):24057–80. doi: 10.18632/aging.202251 (PMC7762479; doi:10.18632/aging.202251)
Supplement: Supplementary Figures [file aging-12-202251-s001.pdf]

SUPPLEMENTARY FIGURES

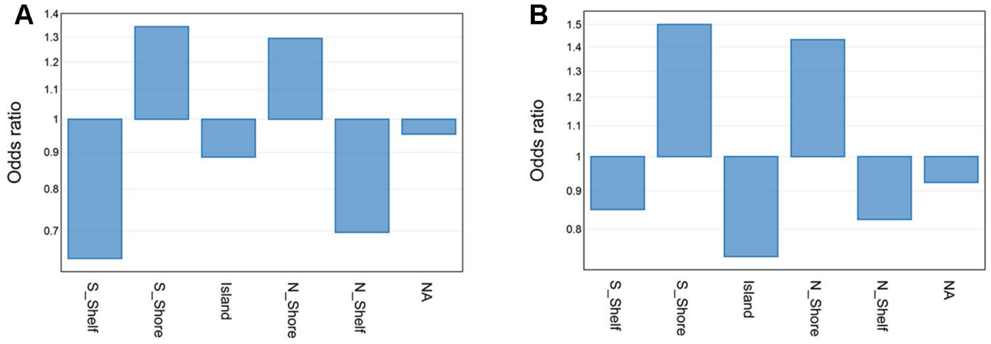

Supplementary Figure 1. Enrichment (odds ratio) of genomic localizations for saDMPs (**A**) and snaDMPs (**B**).

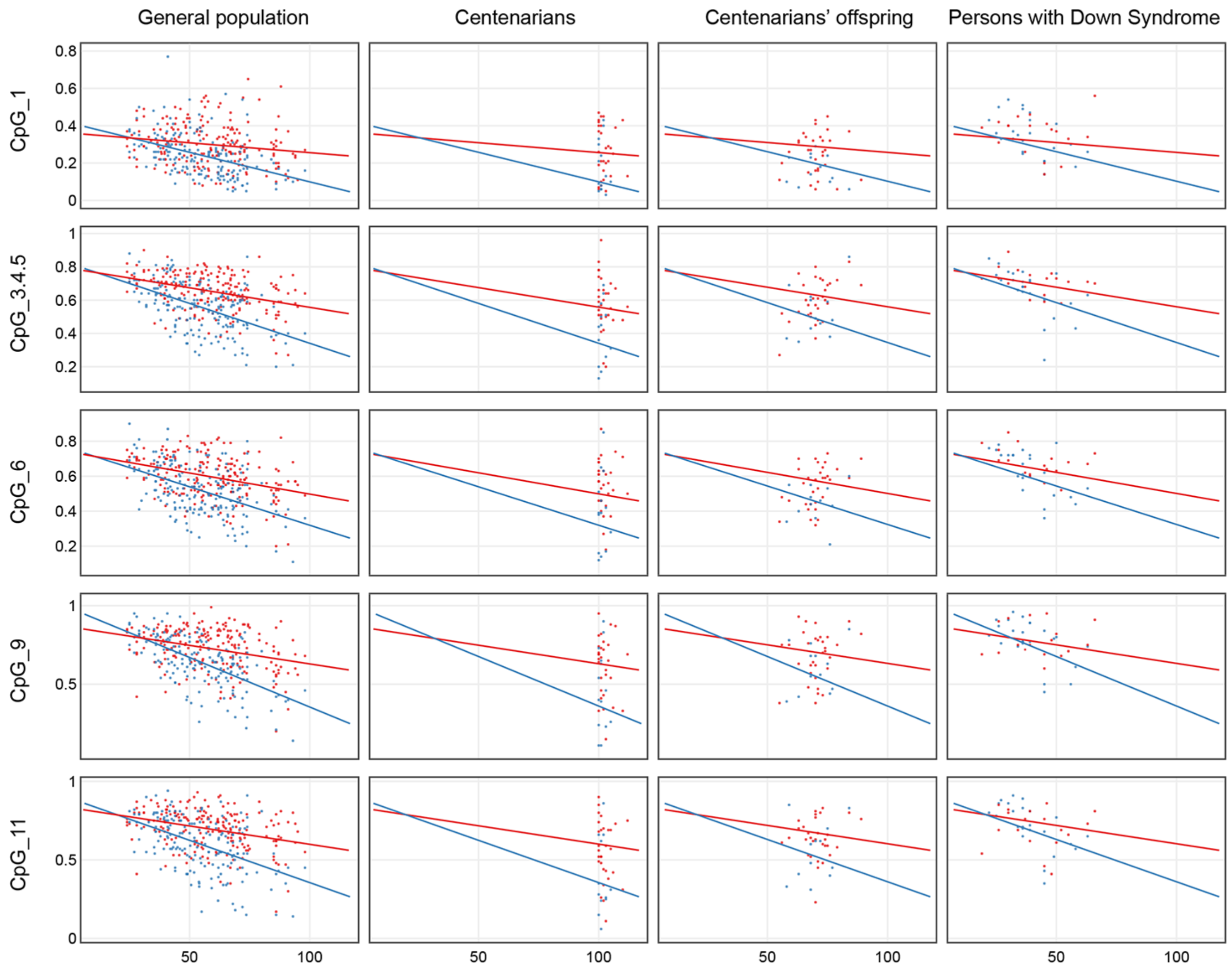

Supplementary Figure 2. Validation of *FIGN* locus by EpiTYPER. For each of the CpG units returned by the EpiTYPER assay, DNA methylation in controls (general population), centenarians, centenarian's offspring and persons with Down syndrome is reported *vs* the age of the subjects. Males are in blue, females are in red. Linear regression between DNA methylation and age was calculated separately for males and females in control subjects and was reported in each plot.

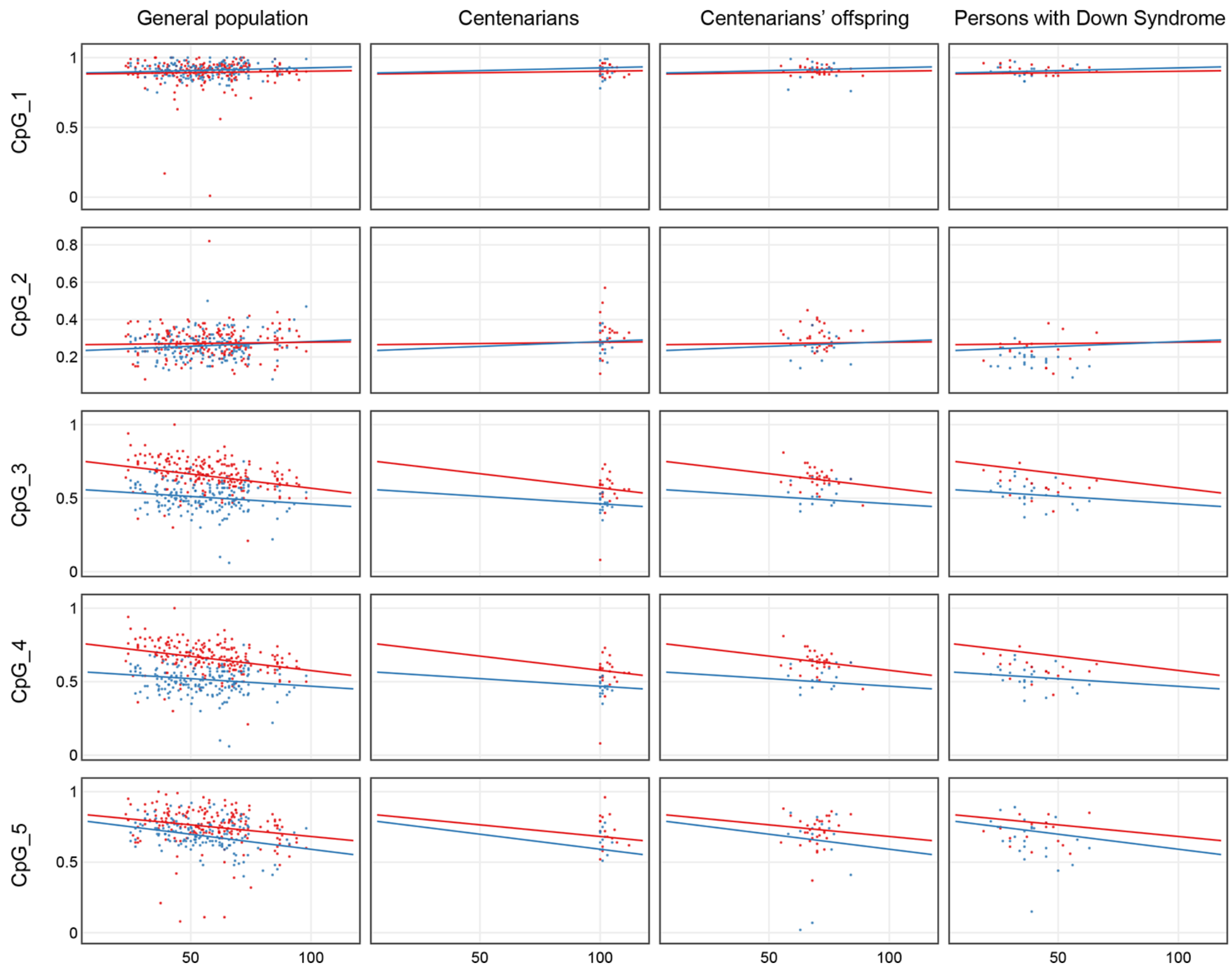

Supplementary Figure 3. Validation of *PRR4* locus by EpiTYPER. For each of the 5 CpG units returned by the EpiTYPER assay, DNA methylation in controls (general population), centenarians, centenarian's offspring and persons with Down syndrome is reported *vs* the age of the subjects. Males are in blue, females are in red. Linear regression between DNA methylation and age was calculated separately for males and females in control subjects and was reported in each plot.

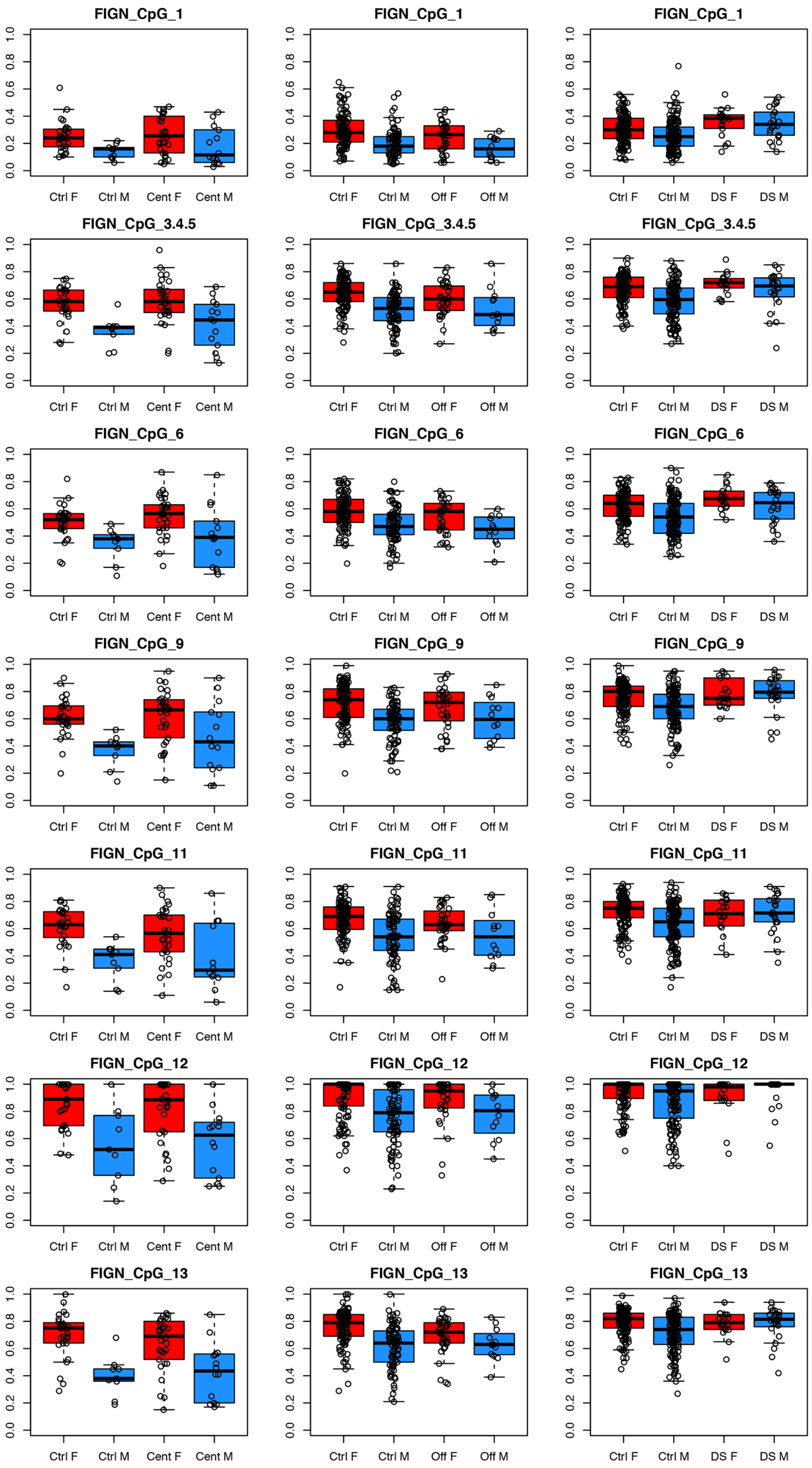

Supplementary Figure 4. Boxplots of DNA methylation for each CpG unit in *FIGN* amplicon in centenarians, centenarians' offspring and Down syndrome cohorts. Left panels: for each CpG unit in *FIGN* locus, boxplots of DNA methylation in male and female centenarians, compared to male and female controls (>80, < 100 years). Middle panels: for each CpG unit in *FIGN* locus, boxplots of DNA methylation in male and females centenarians' offspring, compared to age-matched male and female controls (>54, < 90 years). Right panels: for each CpG unit in *FIGN* locus, boxplots of DNA methylation in male and female Down syndrome persons, compared to age-matched male and female controls (>18, < 67 years).

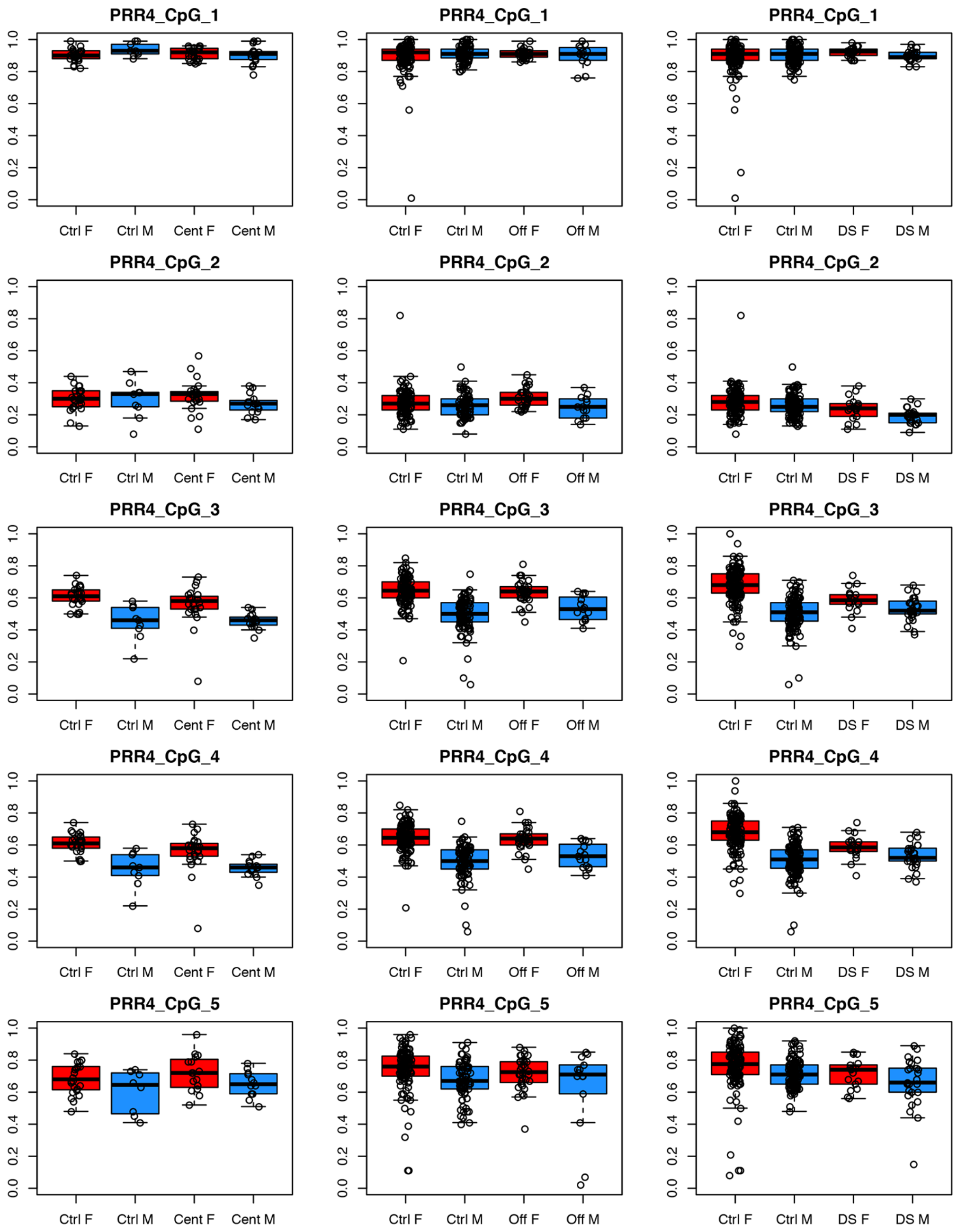

Supplementary Figure 5. Boxplots of DNA methylation for each CpG unit in *PRR4* amplicon in centenarians, centenarians' offspring and Down syndrome cohorts. Left panels: for each CpG unit in *PRR4* locus, boxplots of DNA methylation in male and female centenarians, compared to male and female controls (>80, < 100 years). Middle panels: for each CpG unit in *PRR4* locus, boxplots of DNA methylation in male and females centenarians' offspring, compared to age-matched male and female controls (>54, < 90 years). Right panels: for each CpG unit in *PRR4* locus, boxplots of DNA methylation in male and female Down syndrome persons, compared to age-matched male and female controls (>18, < 67 years).

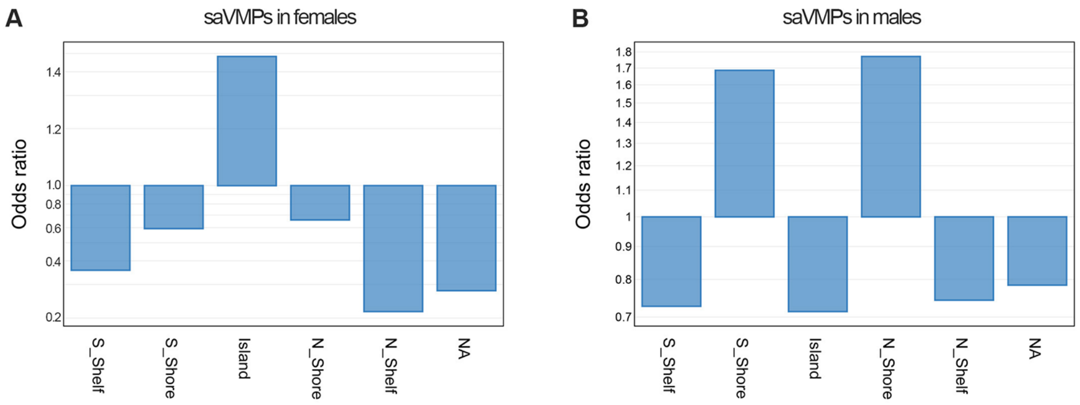

Supplementary Figure 6. Enrichment (odds ratio) of genomic localizations for ssaVMPs calculated from beta values (**A**) or residuals (**B**).

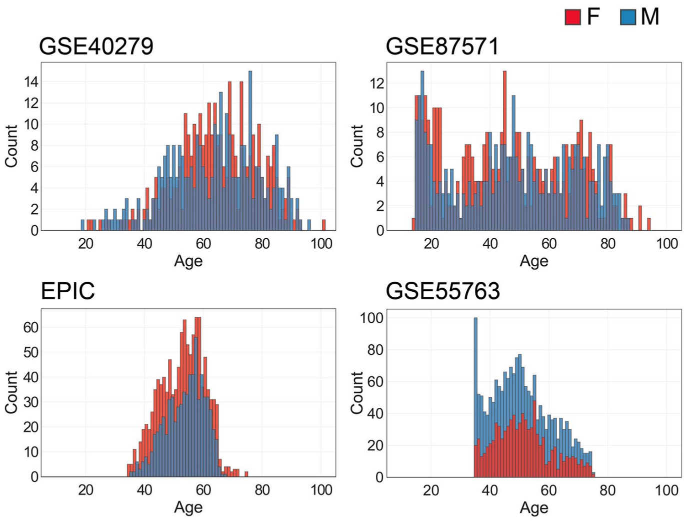

Supplementary Figure 7. Histograms of the number of females (red) and males (blue) according to age in GSE40279, GSE87571, EPIC and GSE55763 datasets.
