# Supplementary material for: Age-related DNA methylation changes are sex-specific: a comprehensive assessment
Source: Aging (Albany NY). 2020 Dec 3;12(23):24057–80. doi: 10.18632/aging.202251 (PMC7762479; doi:10.18632/aging.202251)
Supplement: Supplementary Tables [file aging-12-202251-s002.pdf]

SUPPLEMENTARY TABLES

Supplementary Table 1. Age-by-sex interaction in the number of epimutations and in the values of Shannon entropy. ANCOVA p-values are reported for age, sex and age-by-sex interaction effects.

	Epimutations			Entropy		
	**age**	**sex**	**age:sex**	**age**	**sex**	**age:sex**
GSE40279	6.8093E-08	4.8010E-01	5.1342E-01	7.7160E-01	7.7393E-02	3.6785E-01
GSE87571	9.1517E-20	9.4685E-01	7.2585E-01	1.4711E-17	1.3101E-01	4.7210E-01
EPIC	1.6369E-08	5.8827E-02	8.0821E-02	1.3407E-04	4.8294E-05	1.0310E-04
GSE55763	1.0350E-15	4.9039E-02	2.6199E-02	3.4323E-09	3.2372E-01	1.9585E-01

Supplementary Table 2. Characteristics of the Infinium450k datasets investigated in the present study.

	GSE40279	GSE87571	EPIC	GSE55763
Number of subjects	656	729	1803	2670
Number of females	338	388	1114	860
Number of males	318	341	689	1810
Age range	19–101	14–94	34–74	35–75
Ethnic group (Country)	European and Hispanic (USA)	European (Sweden)	European (Italy)	Indian Asian and European (UK)

Supplementary Table 3. Characteristics of the samples analyzed by the EpiTYPER assay.

	Healthy controls n=419	Centenarians n=49	Centenarians' offspring n=48	Down syndrome n=44
Males	n=198<br>25-98 years	n=15<br>100-105 years	n=15<br>58-84 years	n=25<br>22-63 years
Females	n=221<br>23-98 years	n=34<br>100-112 years	n=33<br>55-89 years	n=19<br>19-66 years

Supplementary Files

Supplementary File 1. Lists of sex- and age-associated differentially methylated positions (saDMPs) and of sex- but not age-associated differentially methylated positions (snaDMPs) resulting from the meta-analysis.

Supplementary File 2. Gene Ontology enrichment of saDMPs and snaDMPs lists.

Supplementary File 3. Probes with significant age-by-sex interaction resulting from the meta-analysis.

Supplementary File 4. Results of the statistical analyses performed on EpiTYPER data on centenarians', centenarians' offspring and Down syndrome cohorts.

Supplementary File 5. Lists of sex-specific age-associated variably methylated positions (saVMPs).

Supplementary File 6. Gene Ontology enrichment of saVMPs lists.

Supplementary File 7. Results of "Ominer" tool for *FIGN*, *DOC2A*, *PEX10* and *PRR4* genes.

Supplementary File 8. Imprinted regions and sex hormones-related genes considered for enrichment analysis.
